# Enhancing questioning skills through child avatar chatbot training with feedback

**DOI:** 10.3389/fpsyg.2023.1198235

**Published:** 2023-07-13

**Authors:** Ragnhild Klingenberg Røed, Gunn Astrid Baugerud, Syed Zohaib Hassan, Saeed S. Sabet, Pegah Salehi, Martine B. Powell, Michael A. Riegler, Pål Halvorsen, Miriam S. Johnson

**Affiliations:** ^1^Department of Social Work, Child Welfare and Social Policy, Faculty of Social Science, Oslo Metropolitan University, Oslo, Norway; ^2^Department of Computer Science, Faculty of Technology, Art and Design, Oslo Metropolitan University, Oslo, Norway; ^3^Simula Metropolitan Center for Digital Engineering, Oslo, Norway; ^4^Center for Investigative Interviewing, Griffith Criminology Institute, Griffith University, Brisbane, QLD, Australia; ^5^Department of Behavioral Science, Faculty of Health Science, Oslo Metropolitan University, Oslo, Norway

**Keywords:** investigative interviewing, child abuse, training, artificial intelligence, chatbot avatar, automatized feedback

## Abstract

Training child investigative interviewing skills is a specialized task. Those being trained need opportunities to practice their skills in realistic settings and receive immediate feedback. A key step in ensuring the availability of such opportunities is to develop a dynamic, conversational avatar, using artificial intelligence (AI) technology that can provide implicit and explicit feedback to trainees. In the iterative process, use of a chatbot avatar to test the language and conversation model is crucial. The model is fine-tuned with interview data and realistic scenarios. This study used a pre-post training design to assess the learning effects on questioning skills across four child interview sessions that involved training with a child avatar chatbot fine-tuned with interview data and realistic scenarios. Thirty university students from the areas of child welfare, social work, and psychology were divided into two groups; one group received direct feedback (*n* = 12), whereas the other received no feedback (*n* = 18). An automatic coding function in the language model identified the question types. Information on question types was provided as feedback in the direct feedback group only. The scenario included a 6-year-old girl being interviewed about alleged physical abuse. After the first interview session (baseline), all participants watched a video lecture on memory, witness psychology, and questioning before they conducted two additional interview sessions and completed a post-experience survey. One week later, they conducted a fourth interview and completed another post-experience survey. All chatbot transcripts were coded for interview quality. The language model’s automatic feedback function was found to be highly reliable in classifying question types, reflecting the substantial agreement among the raters [Cohen’s kappa (κ) = 0.80] in coding open-ended, cued recall, and closed questions. Participants who received direct feedback showed a significantly higher improvement in open-ended questioning than those in the non-feedback group, with a significant increase in the number of open-ended questions used between the baseline and each of the other three chat sessions. This study demonstrates that child avatar chatbot training improves interview quality with regard to recommended questioning, especially when combined with direct feedback on questioning.

## 1. Introduction

Technology is a significant tool for developing new ways to train in educational and practical settings. The use of online learning has expanded tremendously in recent decades, and AI offers teaching and learning solutions in a wide variety of contexts (Cavalcanti et al., 2021, p. 7; Shiohira, 2021). Within the field of child investigative interviewing, finding sufficient opportunities to practice skills and receive feedback, both of which are important in improving skill acquisition and application in practice, is challenging (Lamb, 2016). An important step in increasing such opportunities is to develop dynamic avatars that mimic witnesses in the interviewing context and provide trainees with direct feedback using AI technology. In our ongoing work, we develop conversational child avatars, combining knowledge from developmental psychology, educational psychology, and advanced technology within AI and real-time systems. Early 2-dimensional and 3-dimensional (virtual reality) versions of child avatars that respond dynamically to trainees’ questions have been developed, and stepwise and component-by-component testing are in progress (Baugerud et al., 2021; Salehi et al., 2022; Hassan et al., 2022a,b). In the current study, we are testing a large generative pre-trained transformer (GPT-3), a language model (Brown et al., 2020), as a child avatar chatbot to use for the training of questioning skills in an interviewing context.

The development of automated online resources for practicing interviewing skills is in its infancy. Early digital approaches show promise, such as an eLearning program for professionals used at the Center for Investigative Interviewing. This program, which uses an unrealistic, self-paced child avatar for practicing questioning skills, has been shown to improve interviewing quality in the field over the long term (e.g., Benson and Powell, 2015; Powell et al., 2016; Casey and Powell, 2021). The program uses an option tree structure, presenting a trainee with four questions, from which they are to choose the best option. The child avatar responds with predetermined answers, and the trainee receives feedback about the type of question chosen and whether another would have been better. The self-paced child avatar has also been used as a training tool on its own and has similarly been shown to lead to more desirable questioning (Brubacher et al., 2015). Santtila and colleagues have also developed child avatars for training questioning skills and tested learning effects in combination with pedagogical interventions, such as feedback (process and performance) and behavioral modeling (e.g., Pompedda et al., 2015; Krause et al., 2017; Haginoya et al., 2021). Their results indicate that avatar training, combined with both feedback and modeling, improves interview quality in simulated investigative interviews. More recently, a study that used avatar training with feedback replicated previous findings of increased use of recommended questions by professionals who were conducting both simulated interviews and field interviews with child witnesses (Kask et al., 2022). The trainees formulated questions orally, but until now, the system required an operator to be present to manually code into the software the types of questions asked and thereby to activate a pre-recorded child response (Haginoya et al., 2021). Lately, the group has undertaken a study using an automatized, simulated avatar (Haginoya et al., 2023). In this study, professionals were asked to conduct two child sexual abuse interviews with an online avatar in which the two conditions were with pedagogical intervention (modeling or feedback) or without. The feedback system, which was tested for its accuracy in classifying interviewer questions (as either recommended or non-recommended), showed an overall agreement with manual coders of 72% (chance level being 33%), with a Cohen’s kappa (κ) of 0.49. The results showed improvement in the use of recommended question types (broad and focused invitations, facilitators, directives, and clarifications) in the pedagogical intervention (both modeling and feedback) condition (Haginoya et al., 2023).

Efficiency improvements in interview training tools are needed with respect to availability, time, and cost to achieve the goal of providing sufficient opportunities to receive integrated feedback while practicing interviewing. Reaching this goal is important to counter the consistently poor quality of child investigative interviewing that decades of field evaluation studies have revealed (Cederborg et al., 2013; Johnson et al., 2015; Baugerud et al., 2020). The skills needed to conduct high-quality interviews are complex and require specialized training for their development (Lamb et al., 2002; Powell, 2008; Benson and Powell, 2015; Yi et al., 2016).

Despite the existence of several internationally agreed-upon interview guidelines and their implementation in some organized training (e.g., Lamb et al., 2018; Powell and Brubacher, 2020; Lyon and Henderson, 2021), a significant number of field studies have revealed significant shortcomings in how interviews are conducted. A general finding across these studies is that interviewers do not adhere to best-practice recommendations and ask too many suggestive and closed questions and too few open-ended questions (OEQs). This potentially leads an interviewed child to respond inaccurately and causes the interviewer to unintentionally contaminate the child’s testimony (e.g., Korkman et al., 2008; Johnson et al., 2015; Lamb, 2016; Baugerud et al., 2020). Some researchers have called for evaluating training programs and ensuring that they take into account the principles of human learning (e.g., Powell, 2008; Lamb, 2016; Brubacher et al., 2020). Recent research has indicated that an individual is trained most effectively if the training sessions are spaced out and consist of a complex array of elements, such as theoretical teaching combined with practical assignments, training in relevant contexts, and immediate and detailed individualized feedback during training (e.g., Lamb et al., 2002; Benson and Powell, 2015; Lamb, 2016; Pompedda et al., 2017). It is essential to teach theory-based, yet practically oriented, knowledge that emphasizes key principles and research findings supporting effective interviewing techniques, particularly the persistent use of open-ended questioning with children (Powell, 2008). Training is also more effective when it is followed by opportunities to review and maintain one’s own interviewing skills (e.g., Rischke et al., 2011; Powell et al., 2016; Krause et al., 2017).

In their recent narrative review of thirty studies that assessed the effectiveness of interview-training courses within the police, Akca et al. (2021) found that the majority of them determined that assessed courses had a positive impact both on basic interviewing skills, such as adherence to the protocol that was taught, and on the amount of information elicited from the interviewee. Training was, however, found to be less effective in developing the skill of selecting the best questions to ask. Nine of the 25 studies (36%) that measured questioning style found no training effect. The evidence-based training courses varied in length and intensity [from 1 day to 9 months, with most being a 1 week-long course (37%)] and covered both child and adult forensic interviewing (Akca et al., 2021). Interviewing children requires particular attention to the style and wording of questions (Brown and Lamb, 2015). In one study, Wright and Powell (2006) explored child interviewers’ challenges in adhering to best practice despite extensive training. The interviewers revealed unfamiliarity with the open-question style and experienced difficulties distinguishing between open and closed questions. Experienced, trained interviewers have described the complexity of questioning. The interviewer needs to think one step ahead to formulate the next questions while still staying tuned in to the witness’s answer to the question already asked, not to mention keeping track of the information the interviewer has already obtained and comparing it to existing case information and requirements in penal clauses (Griffiths et al., 2011). This complexity reveals investigative interviewing to be cognitively demanding, a finding supported by recent research that shows that high cognitive load while interviewing is a predictor of low performance (Hanway et al., 2021). In a training context, planning the level of training—such as the asking of basic OEQs or the fine-tuning of skills needed to deal sensitively with reluctance—and designing feedback must be based on principles of human learning and adapted to the trainee’s level of performance (e.g., Powell, 2008; Cyr, 2022; Powell et al., 2022).

Feedback is a crucial component of scaffolded learning, allowing trainees to identify gaps between actual performance and best-practice guidelines and to assess their own learning process (Hattie and Timperley, 2007; Powell, 2008). Immediate and detailed verbal and/or written feedback on question types are found to be effective in online eLearning exercises (e.g., Powell et al., 2016) and within avatar training (e.g., Pompedda et al., 2017; Kask et al., 2022). However, systematic reviews and meta-analyses of educational feedback research (Kluger and DeNisi, 1996; Wisniewski et al., 2020) and of automatic feedback in online learning environments (Cavalcanti et al., 2021) have found feedback to have moderate effects on student learning. A review of 131 studies that, together, comprised 12,000 participants and examined the effect of feedback on performance (across all domains) found that in over a third of cases, the provided feedback negatively affected performance (Kluger and DeNisi, 1996). These results indicate that not all feedback is for the better. A review of 63 studies that looked at automated feedback in online learning environments and its effect on performance in different disciplines found that more than 80% of the studies showed automatic feedback to be as effective as manual feedback and that in just over 50% of the studies, automatic feedback increased performance (Cavalcanti et al., 2021). Successful feedback can be both automated and manual, and successful training is influenced by the feedback design (Henderson et al., 2019; Brubacher et al., 2020). In their study using automated feedback in training with an avatar to increase the likelihood that interviewers pose recommended questions, Haginoya et al. (2023) underline the importance of a high level of accuracy in the question classification model if feedback is to be effective in enhancing learning. Providing feedback on the type of question asked is a common way to support trainees to adhere to recommended guidelines, since assessing questioning is a common way of measuring interview quality. Related to evidence-based criteria for successful feedback design, focusing on types of questions offers a visible measure (best-practice guidelines) to both trainees and educators. Furthermore, trainees, knowing the guidelines, can make sense of the information (type of question) and can act on it in continued training (Henderson et al., 2019). This points to feedback as a process with trainees as active recipients. This aligns with research showing that interview training with feedback must take place over an extended period of time (e.g., Rischke et al., 2011; Cederborg et al., 2021). Additionally, follow-up sessions are needed after the initial training to maintain positive learning outcomes (Powell et al., 2014; Lamb, 2016; Powell et al., 2016). Online learning platforms enable learning to continue over time in properly spaced sessions with recommended rest intervals between them. Online learning also makes refresher courses and self-evaluation exercises possible, both of which are aspects of learning that have been found to maximize the transfer of knowledge and skills in interviewing, ensuring that learning is consolidated (Powell et al., 2010; St Yves et al., 2014; Brubacher et al., 2020). It is known that the possibility of reacting to feedback immediately after receiving it enhances the effectiveness of the feedback given (Henderson et al., 2019). Online learning with training resources, such as a child avatar for practicing interviewing skills, will enable multiple practicing sessions successively.

Practicability is a well-studied advantage of online professional learning. However, for online professional follow-up and refresher training, learner engagement is especially relevant as the learner must maintain the motivation to practice (Powell et al., 2016; Yan et al., 2023). Research on online learner engagement is still in its infancy and deserves consideration when developing and evaluating the efficiency of online learning resources to achieve high learning attainment (Lee et al., 2021). The applications of AI (i.e., machine learning and natural language processing) are being increasingly employed in educational feedback practices to evaluate performance in real time and produce personalized feedback (Wongvorachan and Bulut, 2022). With respect to the current technology, tools for training investigative interviewing skills should aim for automatic and immediate detailed feedback on the core skills, such as questioning.

In this study, we used our large generative pre-trained transformer 3 (GPT-3), a language model (Brown et al., 2020), in a child avatar chatbot for interview training. GPT-3 was finely tuned on child interviewing data that had been coded according to internationally recommended coding standards, using the Standard Interview Method (SIM) Coding Manual (Powell and Snow, 2007; Powell and Brubacher, 2020) and NICHD Investigative Interview coding scheme (Lamb, 1996; Lamb et al., 2007). Fine-tuning enables the language model to respond dynamically to questions posed in an interviewing context, and it enables GPT-3 to classify questions from the interviewer and provide direct feedback during training sessions. The aim of the study was to determine whether training interview skills using a child avatar chatbot, in combination with either direct feedback or no feedback, would demonstrate a learning effect, as measured by the quality of the questions asked in a pre- vs. post-training design. With respect to the conditions listed below, we formulated and sought to test four hypotheses:

1. The effect of the educational lecture plus multiple training sessions: Participants will show increased use of OEQs and decreased use of closed questions after the lecture and across the chat sessions, independent of feedback condition.
2. The effect of direct feedback: Participants receiving direct feedback will ask OEQs to a greater extent than will those receiving no feedback and will pose closed questions less frequently than the non-feedback group.
3. Introducing a delay in training: Participants in the feedback group will show stability in the quality of their interview skills, while those in the non-feedback group will show a decline in interview quality.
4. With respect to post-experience surveys: Participants in the feedback group will rate their overall user experience more positively than will those in the non-feedback group.

Our study further aimed to investigate the degree to which participants were engaged in the chatbot interview activity and the extent to which the chatbot avatar seemed childlike and responded consistently and sensibly. To judge the avatar’s effectiveness as a training tool for practical skills, we measured the extent to which participants experienced the chatbot avatar as useful in gaining knowledge, improving their interview skills, and enhancing their self-efficacy. We also evaluated the reliability of the language model’s coding and classifying of question types (i.e., open-ended, cued recall, and closed questions).

## 2. Materials and methods

### 2.1. Participants

A total of 30 bachelor’s degree students, of whom 27 (90%) were women and 3 (10%) were men (*M_age_* = 25.3, *SD* = 6.4, range 20–50), participated in the study. The participants were students in their first or second year of study in psychology (*n* = 15) or child welfare/social work (*n* = 15). Participants were recruited broadly from among undergraduates in three academic subjects. They were expected to have limited or no prior knowledge or experience of professional communication, such as investigative interviews with children.

### 2.2. Study design and procedure

**Figure 1:**
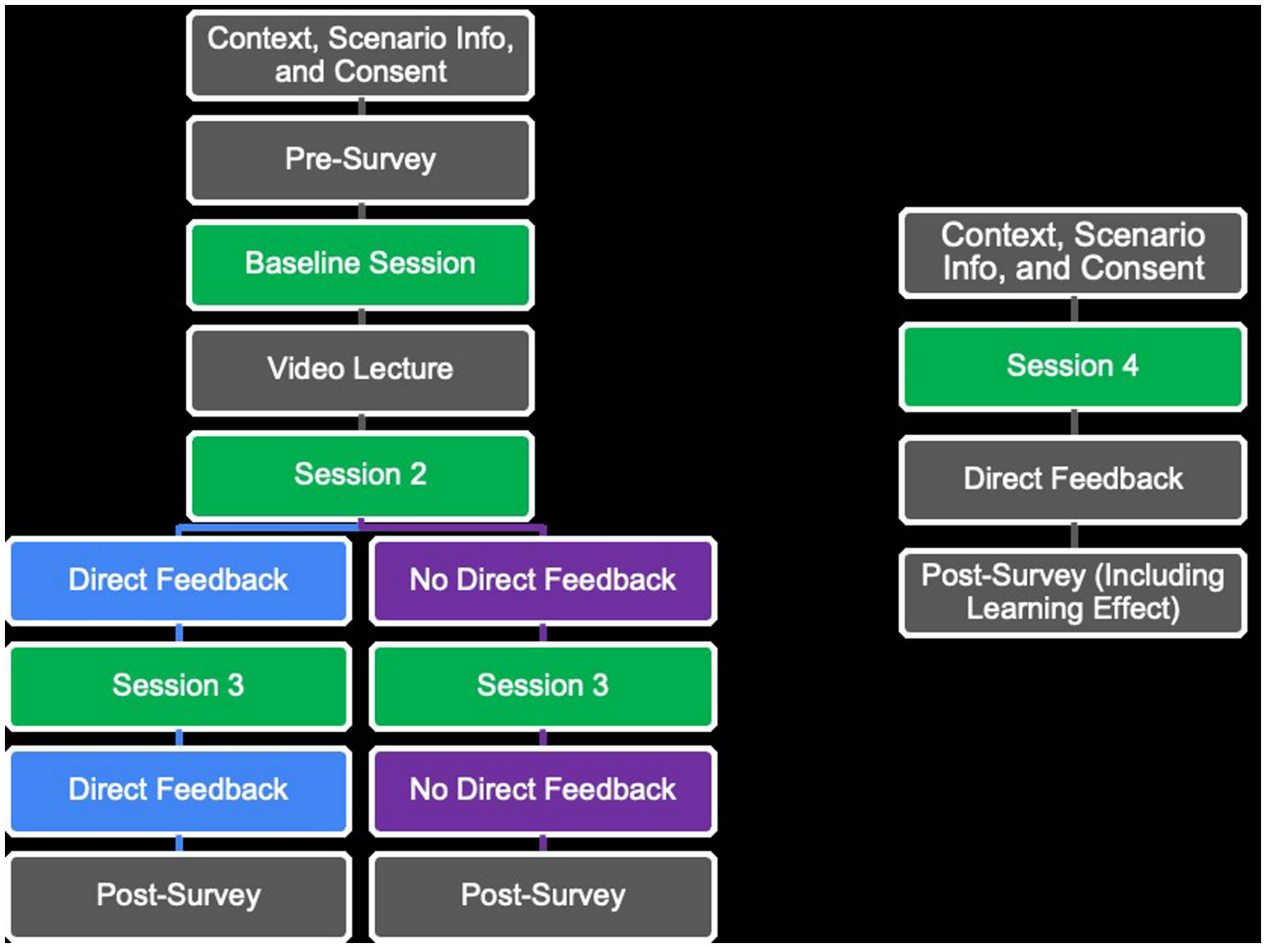
Outline of the study design.

The present study had a 4 (time: four chat sessions; within-subjects) × 2 (non-feedback vs. feedback; between-subjects) mixed design. The participants were randomly assigned to the group receiving direct feedback (*n* = 12) or non-feedback (*n* = 18). An outline of the study design is presented in Figure 1.

Every step of the study was integrated into an online platform. For log in, random ID numbers were generated and distributed by an independent third party at pre-determined time intervals for part 1 and part 2. The link was open for 2 days (24/7) for each part, and participants could participate in the study using their personal computers. Upon entering the platform, they were informed about the study and asked to consent to participation and to the storage of chatbot dialog and feedback for research purposes; they were also told they had the option to withdraw their consent at any time. Participation was anonymous.

The participants were introduced to the context of interviewing and to the case of a 6-year-old girl who was to be interviewed about alleged physical abuse. A short pre-survey about non-identifiable demographic information was run before conducting the first chat session (baseline). After conducting the first chat session, all the participants received a 23-min educational lecture on memory, witness psychology, and recommendations for how to question children before chat session 2. They conducted a total of four chat sessions lasting 7 min each, with a week’s delay between chat sessions 3 and 4. After chat sessions 2 and 3, one group (*n* = 12) received direct feedback, whereas the other group (*n* = 18) did not receive feedback. Post-experience surveys were given at the end of part 1 (after chat session 3) and part 2.

The participants took a total of approximately 90 min to complete part 1 and part 2. Information explaining the study (except for the initial information letter) and the chatbot was offered in English, and the participants were required to write their questions and responses in English. Three chat session dialog were in Norwegian as the participants wrote their prompts/questions in Norwegian, with the language model replying in the corresponding language. The participants rated their English proficiency on a 5-point scale, from novice to expert, yielding a reporting average of 3.06 (*SD* = 0.96). Each participant received a 200 Norwegian kroner (NOK) gift card for participating voluntarily.

### 2.3. Materials

#### 2.3.1. Training the chatbot avatar

The GPT-3 (Brown et al., 2020) is a powerful generative model that has been trained on a large corpus of online data and can also be fine-tuned for specific tasks, such as child interviewing. The point of using this model in training child interviewing is to practice on eliciting accurate and coherent information about self-experienced events. By fine-tuning GPT-3 on various personas and stories, the language model can engage in dynamic dialog, even within the context of investigative interviews. This means that the child avatar’s responses are dynamically generated by the language model, rather than having merely been pre-recorded. However, to accomplish this, the GPT-3 language model must be fine-tuned with relevant data from interviews with children. This is crucial to facilitate realistic dialog, which involve turn-taking, and in particular to credibly mimic age-appropriate child behavior.

**Figure 2:**
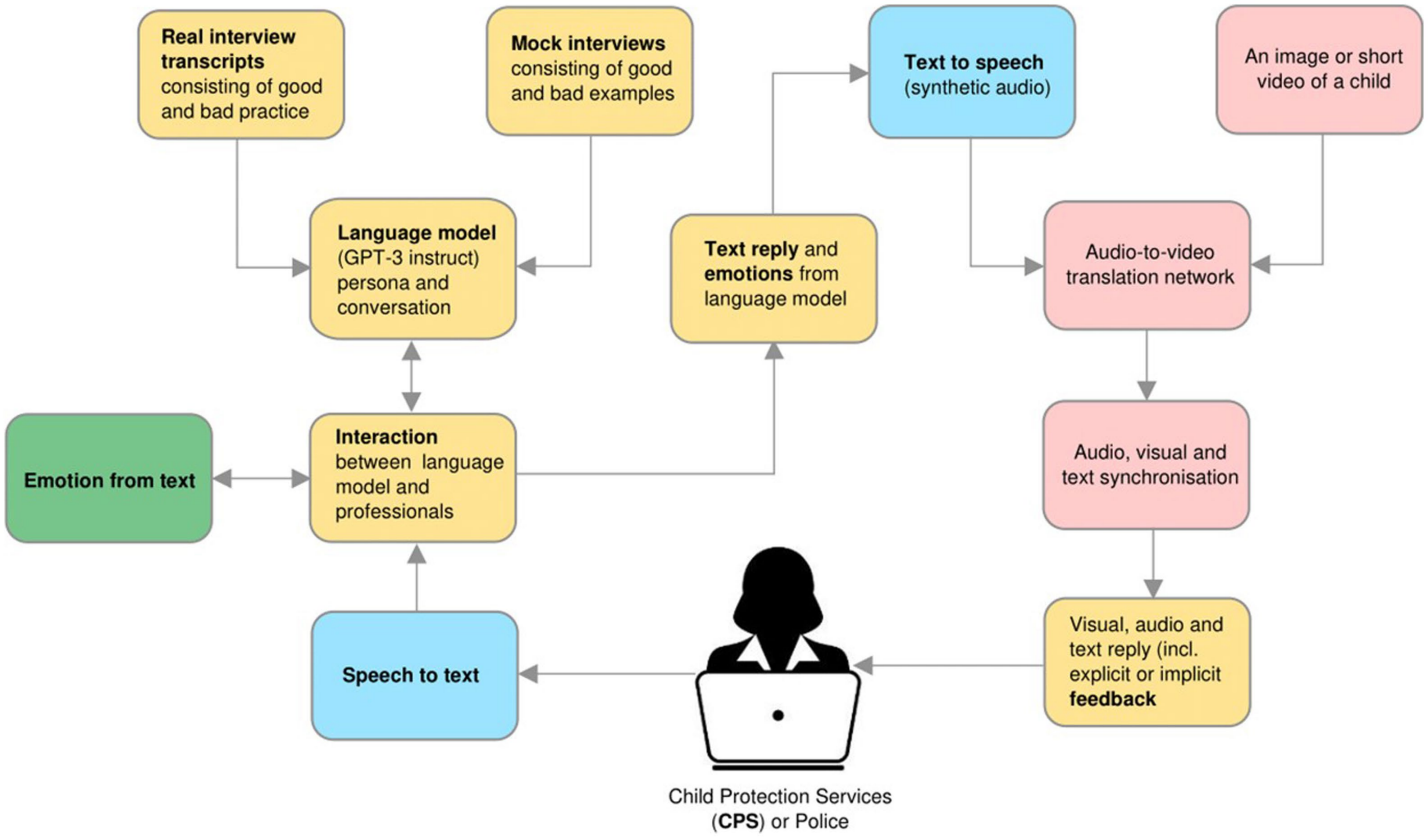
Outline of the system architecture. Green blocks denote the interactive parts, yellow blocks are related to language, blue are related to audio, and pink are related to visualization (Salehi et al., 2022, p. 7).

The language model has been trained using coded transcripts of well-designed mock investigative interviews provided by the Centre for Investigative Interviewing (Powell et al., 2016). Coding, which was performed by two of the authors and two research assistants, reached substantial agreement among the raters with Kappa coefficients (*κ*) of 0.70 or higher. Following best-practice guidelines using the SIM and the corresponding coding manual, the categories used were open-ended (i.e., initial invitation, breadth, depth, and specific directives), minimal encouragers (facilitators), cued recall (wh-), option-posing, and leading (suggestive; Powell and Snow, 2007; Powell and Brubacher, 2020). For categorization of suggestive questions, the NICHD Investigative Interview coding scheme was used due to the training of the language model (Lamb, 1996; Lamb et al., 2007). The training data comprised 700 transcripts of well-designed mock investigative interviews conducted by professional trainees (police, psychologists, and social workers) in a training context in which a trained actor played the allegedly abused child. The trained actor was a research assistant with content knowledge. In addition, they participated in a four-stage training, in which the person was taught to provide the right stimuli in the role of the child and thereby provide effective feedback (for a detailed description, see Powell et al., 2022). By “well-designed,” we mean that the actor gave indirect feedback through the pattern of responses; for example, when the trainees asked the desired OEQs, the child actor responded by giving forensically relevant information. On the other hand, when the trainees asked closed or leading questions, the actor responded with a brief “yes” or “no.” Using such data to fine-tune the language model enabled GPT-3 to recognize the patterns of reinforcement in response to recommended questions. The model responded the same way as when a child would be interviewed. The mock interviews were based on multiple scenarios of alleged sexual and physical abuse toward young children of both sexes. Scenarios of both male and female perpetrators committing intra-and inter-familial abuse were among the mock interview material, making it possible to use different scenarios with a variety of case characteristics when conducting other studies testing various components and developed prototypes. See Figure 2 for the system architecture.

To develop the storyline for the present study, an anonymized and partly fictitious transcript of suspected physical violence was created manually using real investigative interview transcripts. Then, similar cases were selected from mock-interview transcripts and used to fine-tune the GPT-3 model for the current case scenario. This was in addition to the mock interviews used for basic training. To optimize the GPT-3 Davinci model for the chatbot application, we employed a fine-tuning process to enhance its performance in downstream tasks. The fine-tuning involved adjusting key hyperparameters, which included setting the batch size to 1, utilizing a learning rate multiplier of 0.1, running the model for four epochs, and assigning a prompt loss weight of 0.01. To facilitate this fine-tuning, 46 prompt and completion examples were used, allowing the model to understand better and adapt to the specific requirements of the chatbot application.

#### 2.3.2. Interventions

##### 2.3.2.1. Instructions with case scenario

**Figure 3:**
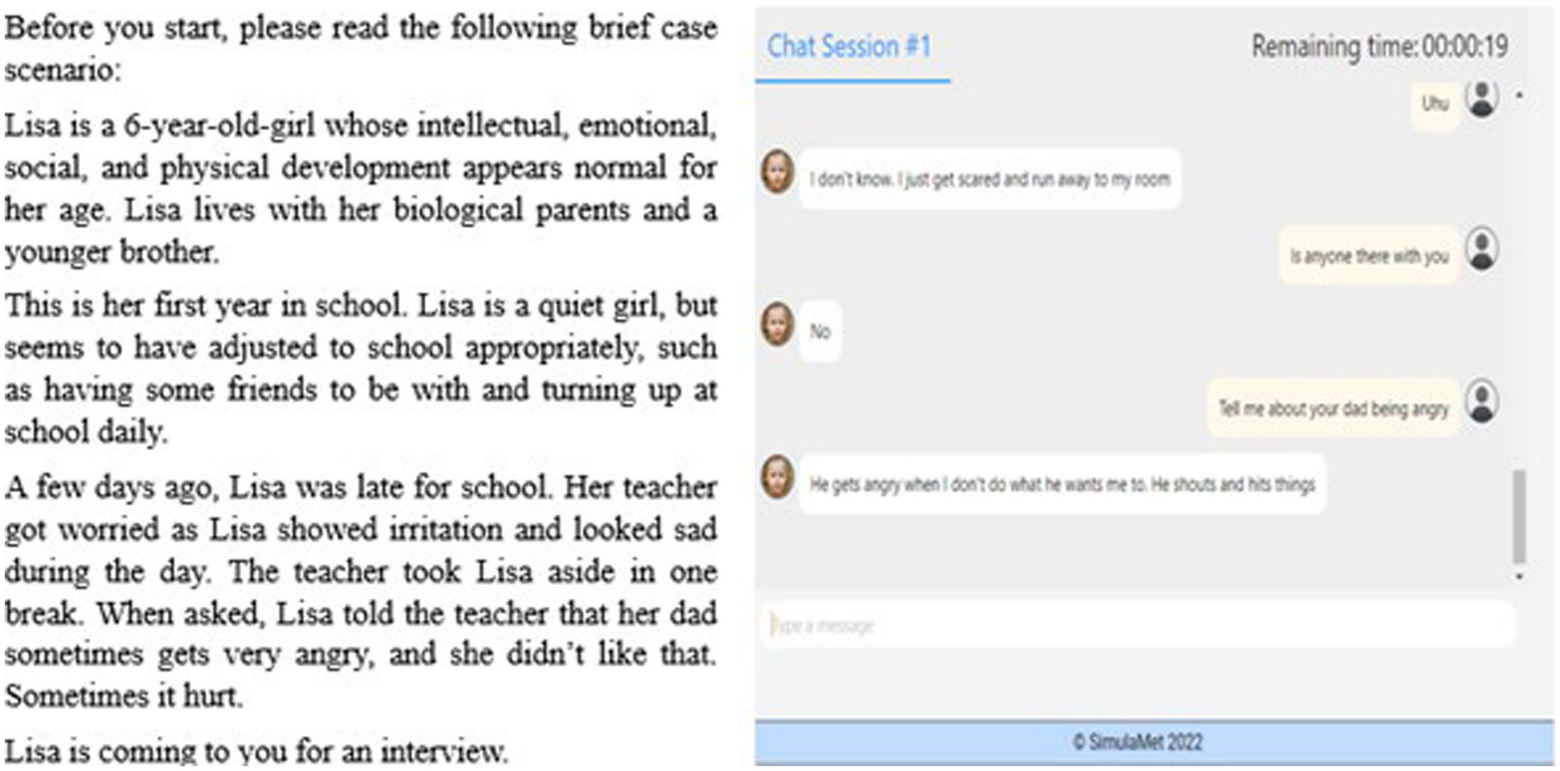
Screenshot of **(A)** the case scenario and **(B)** an example string of the chatbot dialog.

We chose the scenario of alleged intrafamilial physical abuse with the understanding that our participants were students with little or no known experience of interviewing or working within the area of child abuse and neglect. The scenario is presented below in Figure 3A. Prior to the chat session, the participants were given a brief introduction explaining what they would be asked to do:

In this study, you meet Lisa, a 6-year-old girl. You will be requested to interview her about an alleged event with the aim of eliciting accurate information about what she has experienced. See the scenario below. The interview focuses on the part of the interview where the child, the alleged victim, is encouraged to describe the incident under investigation. Your questioning should last about 7 minutes.

This introduction also offered suggestions about how to start the interview, including asking some questions about the child’s name and interests, even if the focus of the exercise was to elicit from the child what had happened. Suggesting the initial question(s) conforms to the approach used in the online child avatar used for training purposes at the Centre for Investigative Interviewing (Powell et al., 2016). This study was a text-only design, producing text strings and providing a small picture of a girl, aged 6, on the left-hand side. The image of the girl was generated using StyleGAN (Karras et al., 2019), a state-of-the-art generative adversarial network (GAN) developed by Nvidia. Figure 3B shows an example string. When the chat session started, a display of the chat log appeared, followed by a white field with the words “type a message.” After typing something, the participants pressed Enter, and the child avatar response appeared word for word on the left side.

##### 2.3.2.2. Educational lecture after baseline chat session

One of the authors, a clinical psychologist specializing in developmental and trauma psychology, pre-recorded a 23-min lecture. The lecture included an introduction to human memory and witness psychology, focusing on implications for questioning children about an alleged event. The topics included were autobiographical memory; memory as a constructive activity being vulnerable to, for example, forgetting, misattribution, and persistence; memory of traumatic events; and the main principles underlying “best-practice” questioning in child investigative interviews.

##### 2.3.2.3. Direct feedback

**Figure 4:**
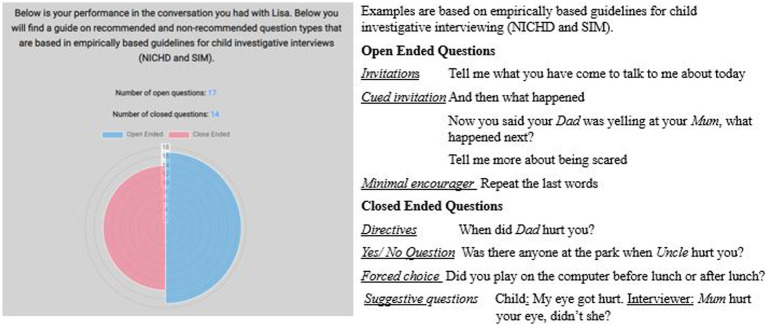
Screenshot of **(A)** the visualized feedback and **(B)** educational feedback with examples of open-ended and closed questions. The feedback given after the first chat session is shown.

Automated feedback in an online interview training system must be integrated, immediate, and highly accurate on an individual level. Direct feedback in the current study was provided to one group of participants by showing each of them the number of OEQs and closed questions asked during the previous chat session, presented as the total of open and closed questions. In addition, the number of questions asked were highlighted with different colors in a polar area chart (see Figure 4A). After this summary, educational feedback in text form was provided to show examples of open-ended and closed questions within the basic categories listed in empirically based guidelines from the NICHD and SIM (Lamb, 1996; Lamb et al., 2007; Powell and Snow, 2007; Henderson et al., 2019; Powell and Brubacher, 2020). The examples were not directly related to the case scenario but were chosen to illustrate the different question types. New examples were given after each chat session (see Figure 4B).

Indirect feedback was provided to all the participants by the language model, which was trained on well-designed mock interviews that built on the learning principle of reinforcement of desirable behavior. When open-ended questions and open directives were posed, the trained language model generated child responses with relevant and extensive information. When closed-ended and suggestive questions were asked, the model generated non-responsive replies to indicate to the trainee that these question types are not productive. (An evaluation of whether or not the language model provided indirect feedback systematically, as in mock interviews, is outside the scope of this paper).

##### 2.3.2.4. Coding the chat interviews

Transcripts from the chat sessions consisted of a total of 120 dialogs. These were coded for question type using the coding manual for the SIM and NICHD (Lamb, 1996; Lamb et al., 2007; Powell and Snow, 2007; Powell and Brubacher, 2020). Coding was conducted turn by turn with a total of 14 categories of question types (see Table 1). One turn referred to one interaction, which comprised one interview question and one child avatar response. Kappa coefficients were calculated to assess inter-rater reliability, with *κ* of 0.70 indicating substantial agreement among the raters. The chat interview dialogs were coded into non-substantial and substantial phases in accordance with the phases in best-practice guidelines for child investigative interviews. To be coded as a substantial phase, the dialog must explicitly be about or request information directly relevant to the case in question. The generated child responses were also coded but will not be referred to further here.

**Table 1:** Descriptions and examples of question types in the terminology of the SIM and NICHD.

SIM terminology	Description	Sample questions
Initial invitation [open]	Open questions encourage elaborate and coherent responses without specifying the information being sought.	Start from the beginning and tell me everything…
		Tell me everything that happened.
Breadth prompt [open]	Invitation—tell as much as possible.	Then what happened…?
	Breadth—ask for more information about the event in general.	What happened next?
Depth prompt [open]	Depth—request more information about something already mentioned.	Tell me about the part where (verb/ action).
		What happened when X…?
Descriptive [open specific]	Either an event or action or, more specifically, an object, person, or location.	Tell me more about X…
		You mentioned a shelter. Tell me all about the part when you saw the shelter.
Minimal encourager (ME)	Support the narrative to continue	Uh huh, mm-hmmm.
		(Repeat last words said.)
Specific cued recall	These questions limit the response by specifying the expected information.	What color? Where? When? etc. Who was at home when you got hit?
Specific yes/no [closed]	Closed questions limiting the responses. Ask the interviewee to recognize information (as opposed to recall).	Did it happen at night?
		Was it a man?
Specific forced choice/option posing [closed]	These questions specify the expected content of the response.	Were you standing or sitting?
	Forced-choice questions present or imply options to choose among.	Did she have clothes on or not?
Leading types of Qs:	Leading questions suggest a certain response is the desired one or introduce information not previously mentioned or otherwise established without giving the interviewee the opportunity to deny the information.	There was a red carpet there, wasn’t there? Grandpa forced you to touch him, did not he?
Repeated question		
Leading		
Pressure		If you hurry up, we’ll be done soon!
Referring		Your teacher told me…
Indication		Why do you think she did that to you?
Visual		

##### 2.3.2.5. Post-experience survey

After completing part 1 and part 2 of the study, all the participants were asked to fill out a post-experience survey designed to measure aspects of their experience with the avatar chatbot. Communicative intention was used as a guiding principle in choosing criteria for each aspect we measured. We also took into account the context and aim of the investigative interview, inspired by the guidelines of Rapp et al. (2021) and the field’s lack of agreement as to how to best measure these aspects. Assessing engagement, we chose to use flow items from the Game Experience Questionnaire (IJsselsteijn et al., 2013) and one additional item associated with involvement within educational literature (Rapp et al., 2021). The overall quality of experience (QoE) was assessed using one indicator (Alben, 1996). To keep the surveys concise, we chose three to four items to assess conversational qualities related to the realism (childlikeness) and consistency and sensibility (appropriateness) of the child avatar’s responses (see Table 1 for an overview of the items; Wilson et al., 2018; Rapp et al., 2021; van der Lee et al., 2021). To judge whether the chatbot avatar offered an efficient training tool, we asked two statements about the learning effect at the end of the post-experience survey, part 2. The concept of self-efficacy (Bandura, 1978) refers to one’s belief in one’s own abilities to perform in a specific context (in this instance, child investigative interviewing). The degree of self-efficacy is considered to imply the integration of skills and their transfer from training to actual practice (Nørgaard et al., 2012). A 5-point Likert scale (strongly disagree—strongly agree) was used (see Table 2 for all items). At the end of the study, the participants were asked two OEQs that targeted their opinions about the use of the chatbot avatar for learning purposes and their suggestions for improvement. They are not included in this study.

**Table 2:** List of post-experience survey items used after part 1 and part 2.

**Overall quality of experience**
How was your overall experience talking to the chatbot?
**Engagement**
It felt like the interview took ages.
I felt completely absorbed in the interview.
I forgot everything around me.
**Realism**
I felt like talking to a child.
The interview had a natural turn-taking.
I could imagine the experience the child talked about.
**Consistency**
The child’s responses negated each other.
The child’s responses were consistent with the child’s story.
The child’s responses were random.
The child’s responses were specific.
**Sensibility**
The child’s responses took an unexpected turn.
The conversational responses from the child were appropriate.
The child’s responses made no sense.
The child’s responses were sensible.
**Learning**
Using the chatbot avatar aids me in acquiring knowledge and skills within questioning.
Practicing with the avatar can enhance my self-efficacy.

### 2.4. Statistical analyses

To validate the automatic coding of question type conducted by the classification model (the fine-tuned GPT-3), the inter-rater reliability between the model and the human raters (see section 2.3.2.4 Coding of Chat Interviews) was computed running Cohen’s kappa (κ). The coded chat session interviews were analyzed using a one-way repeated measures ANOVA on the data in the substantial phase to test if the participants, independent of condition, showed an increase in the number of OEQs asked and a decrease in the number of closed questions. We conducted mixed ANOVAs with the Bonferroni *post hoc* analysis with pairwise comparisons to investigate further and test the second hypothesis. We expected the participants in the feedback condition to show greater improvement in the use of OEQs and to pose less closed questions compared to those in the non-feedback condition. Paired samples t-tests were used to test the third hypothesis related to the effect on questioning between chat sessions 3 and 4 associated with a week’s delay. A mixed ANOVA assessing the QoE, and Independent sample T-tests regarding user engagement were computed to analyze data from the post-experience surveys.

#### 2.4.1. Preliminary analysis

All data were analyzed using SPSS, version 28. Preliminary exploratory analyses did not identify significant outliers on any variables in relation to the pattern of questions asked. For this purpose, open-ended questions (invitations, breadth, and depth prompts and descriptive) were collapsed into one category—OEQs. Furthermore, yes/no and forced-choice (also called option-posing) questions were collapsed together with leading questions into higher-level category closed questions. Cued recall (wh-) and minimal encouragers (ME) were held as individual categories in the analysis related to assessing the learning effect.

### 2.5. Ethics

The study received approval from the Norwegian Agency for Shared Services in Education and Research (SIKT), project number 614272. No personal data were stored during the data collection.

## 3. Results

### 3.1. Validation of the automatic coding

To determine whether to expect an effect of the direct feedback, the automatic coding function conducted by the classification model was assessed. The level of agreement between the classification model and human coders was established using data with 3,206 turns from the chat sessions (including those produced by students who withdrew from the study) to run an inter-rater reliability test for the three higher-level categories: OEQs (including ME), closed questions, and cued recall (wh-questions). Cued recall, the third category, can be placed along a continuum from open-ended to closed without clearly defined boundaries (Lyon, 2014). These questions have been classified differently across research groups (Brubacher et al., 2020). Our analysis showed an 86.7% agreement for the OEQs, 85.3% for the closed questions, and 87.4% for the cued recall questions, with an overall Cohen’s κ = 0.80. The participants received direct feedback in the categories of open-ended and closed questions, as coded by the language model. Cued recall questions were not included in the direct feedback in the current study.

### 3.2. Learning effects across sessions and feedback condition

The participants conducted four chat sessions, each producing 120 transcripts with a total of 2,711 turns, 43.2% (1,170 turns) of which were in the non-substantial phase of the chat session and 56.8% (1,541 turns) in the substantial phase. We used data from the substantial phase to engage in further analyses.

#### 3.2.1. Initial analysis

The first analysis was conducted on the sample as one group. A one-way repeated measure ANOVA was conducted to inspect our first hypothesis, in which we would see an increase in the use of OEQs (ME not included) and a decrease in closed questions after the lecture and across chat sessions (Time 1–4). For OEQs, there was an overall significant main effect of chat sessions; *F*(3, 27) = 9.02, *p* < 0.001, partial ƞ^2^ 0.50. There was no significant effect across chat sessions on the frequency of use of closed questions. This result partly supports our first hypothesis, that is, that we would find an effect of educational intervention and three training sessions on the frequency of use of OEQs. However, the expected decrease in the posing of closed questions was absent.

#### 3.2.2. Open-ended questions

To investigate the effect of receiving feedback vs. no feedback across the four chat sessions on the number of OEQs used, a mixed ANOVA was conducted with the feedback condition (between-subject) and chat sessions (1–4; within-subject) as independent variables and number of OEQs asked as dependent variables. Descriptive statistics are presented in Table 3.

**Table 3:** Effect of training (chat session 1–4) and feedback condition on asking open-ended questions.

Time chat sessions			
	Participants (*N*)	Sum	Mean
1 Baseline non-feedback	18	10	0.6 (1.2)
Baseline feedback	12	6	0.5 (0.8)
**2 Post-lecture and training**			
Non-Feedback	18	29	1.6 (2.2)
Feedback	12	36	3.0 (3.0)
**3 Post-training**			
Non-Feedback	18	25	1.4 (1.7)
Feedback	12	51	4.3 (4.3)
**4 Post-training-delay**			
Non-feedback	18	22	1.2 (1.3)
Feedback	12	37	3.1 (2.6)

Values in parentheses are standard deviations.

A descriptive analysis showed standard deviations that could indicate that the number of questions asked by one or more participants in the feedback condition had high values. However, preliminary analysis identified no significant outliers.

Conducting the mixed ANOVA, we found that Mauchly’s test of sphericity was violated; due to a high estimated epsilon (>0.75), we used the Huynh–Feldt correction for sphericity (Field, 2013). A significant main effect of time, that is, chat sessions, emerged with *F*(2.49, 69.89) = 7.75, *p* < 0.001, partial ƞ^2^ = 0.22, in addition to a significant main effect of the feedback condition of *F*(1, 28) = 7.34, *p* < 0.01, partial ƞ^2^ 0.21, and a significant interaction between time and feedback condition. More specifically, the results revealed a significant difference in the OEQs asked over the chat sessions between the two intervention groups: *F*(2.49, 69.89) = 2.90, *p* < 0.05, partial ƞ^2^ 0.09. This indicates a significant difference in performance between the feedback vs. non-feedback groups in relation to the use of OEQs. We next investigated where the significant differences occurred across the four training sessions for each feedback condition.

##### 3.2.2.1. Feedback condition

A *post hoc* analysis with a Bonferroni adjustment revealed a significant difference in the number of OEQs posed between chat session 1 (baseline) and chat session 2 (2.5, 95% confidence interval (CI) [0.62, 4.38], *p* = 0.005). A significant difference was also found between chat session 1 and chat session 3 (3.75, 95% CI [1.49, 6.01], *p* < 0.001) and between chat session 1 and chat session 4 (2.58, 95% CI [0.9, 4.19], *p* < 0.001). There were statistically significant differences, with an increase in the number of OEQs asked by the participants in the feedback condition between the baseline and each of the other three chat sessions. Between the other chat sessions (2, 3, and 4), there were no significant differences in the use of OEQs.

##### 3.2.2.2. Non-feedback condition

A *post hoc* analysis revealed no significant differences in the number of OEQs asked by the participants between any two pairs of the four training sessions.

**Figure 5:**
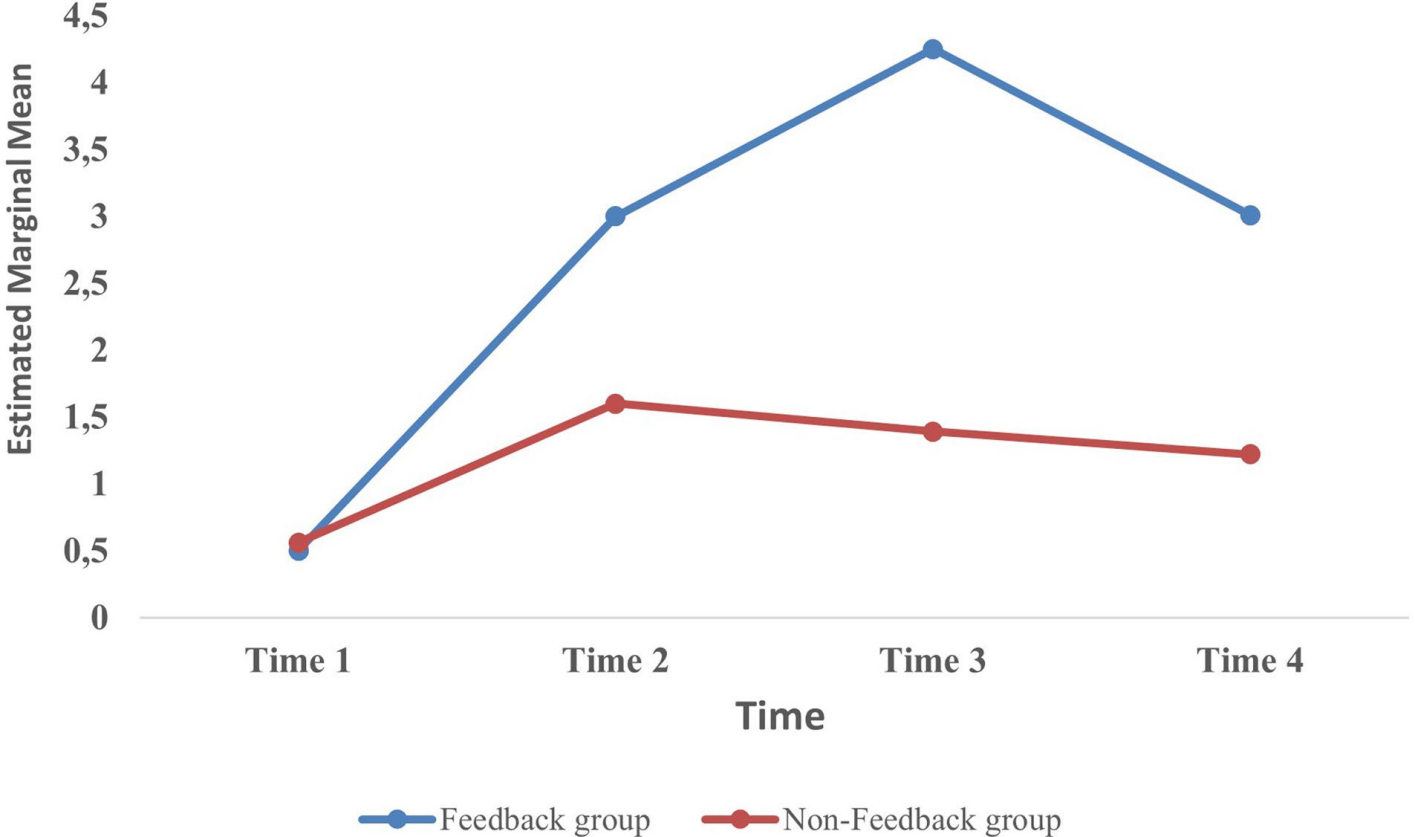
Mean number of open-ended questions asked across four sessions in the feedback and non-feedback groups.

Investigating the interaction effects of the chat sessions and feedback vs. non-feedback intervention, a *post hoc* analysis with a Bonferroni correction revealed significant mean differences between the participants in the feedback and non-feedback groups in chat session 3 (2.86 (95% CI [0.59 to 5.14], *p* = 0.016)) and chat session 4 (1.86, 95% CI [0.39 to 3.33], *p* = 0.015). In chat session 2, no significant differences between the two groups were revealed. In sum and consistent with our second hypothesis, analyses revealed that the participants who received direct feedback asked more OEQs than those who did not receive feedback. The results showed a statistically significant increase in the use of OEQs between chat session 1 (baseline) and each of the other three chat sessions in the feedback condition. There were no significant differences in the number of OEQs posed among the participants in the non-feedback group. Furthermore, the results showed a significant difference in the number of OEQs asked by the participants in the feedback group compared to the non-feedback group in chat session 3 and chat session 4. See the interaction graph in Figure 5 for an illustration of the development in the mean number of OEQs asked.

#### 3.2.3. Minimal encouragers, cued recall, and closed questions

A 4 (chat sessions, within-subjects) x 2 (feedback vs. non-feedback, between-subjects) mixed ANOVA was conducted to explore the effect of training on minimal encouragers (ME), cued recall (wh-) questions, and closed questions (that is, yes/no, forced-choice, and leading questions). No statistically significant effects were found for any of these categories. Our first and second hypotheses expected a decrease in the use of closed questions and were not supported. For the percentage of total questions asked per question type at the baseline (chat session 1) and in the following three chat sessions, see Table 4.

**Table 4:** Percentage of questions asked in chat sessions 1 to 4: minimal encouragers, cued recall, and closed questions.

Time chat sessions			Minimal encouragers		Cued recall		Closed questions	
		Participants (*N*)	Sum	Percent of total	Sum	Percent of total	Sum	Percent of total

1 Baseline non-feedback		18	27	16.5%	62	14.8%	80	13.9%
Baseline feedback		12	10	9.4%	34	8.1%	54	8.8%
**2 Post-lecture training**								
Non-feedback		18	47	20.6%	63	15.1%	89	14.6%
Feedback		12	51	27.9%	52	12.4%	49	8.0%
**3 Post-training**								
Non-feedback		18	49	21.1%	66	15.8%	96	15.7%
Feedback		12	45	21.3%	61	14.6%	56	9.2%
**4 Post-training delay**								
Non-feedback		18	28	15.0%	33	7.9%	107	17.5%
Feedback		12	54	24.4%	47	11.2%	80	13.1%
		Total	311		418		611	

Regarding the types and frequency of questions asked in the chatbot dialogs in each of the four training sessions, only the number of OEQs changed significantly with respect to how many were posed. The participants in the feedback condition asked significantly more recommended questions than the participants who received no direct feedback during training. The frequency of non-recommended questions showed no significant change.

#### 3.2.4. Effect of 1-week delay

Paired samples *t*-tests were conducted to evaluate the mean use of question types in chat session 3 and, after a week’s delay, in chat session 4 in the feedback and non-feedback conditions. For the group receiving direct feedback, the analysis revealed no significant change in the number of questions asked in any of the main categories, that is, open-ended, cued recall, minimal encouragers, and closed questions between chat sessions 3 and 4. Similarly, no significant change in the posting of different question types was found for the non-feedback group.

In sum, these findings support our hypothesis of a stability in the pattern of questioning in the feedback condition. At the same time, the finding that the participants in the non-feedback condition did not change their pattern of questioning between sessions 3 and 4 did not support the third hypothesis as we expected a decrease in OEQs and an increase in closed questions in this group.

### 3.3. Post-experience surveys

Our results from the post-experience surveys administered after the participants concluded part 1 of the study (after three chat sessions) and, following a week’s delay, after they completed part 2 (after the fourth chat session) were analyzed next. The scale used was a 5-point Likert scale (strongly disagree—strongly agree).

#### 3.3.1. User experience aspects

QoE refers to the user’s subjective evaluation of various aspects of the interaction with the chatbot avatar. These aspects include the avatar’s ability to serve the intended purpose and the subjective experience of being engaged. Conducting a mixed ANOVA, no significant difference between how the participants in the two feedback conditions evaluated the overall experience of chatting with the child avatar was found. Descriptive statistics are presented in Table 5. Our fourth hypothesis was thus not supported.

**Table 5:** Mean and standard deviation values regarding quality of experience and engagement in the feedback and non-feedback groups after part 1 and part 2.

	Feedback		Non-feedback	
	*n* = 12		*n* = 18	
	Mean	SD	Mean	SD
QoE part 1	3.58	0.90	3.39	0.61
QoE part 2	3.25	0.87	3.44	0.78
Engagement part 1	3.73	0.60	3.95	0.72
Engagement part 2	3.59	0.85	3.65	0.80

Engagement refers to the participant’s feeling of being absorbed by and experiencing the flow while engaging in the exercise. It is considered especially relevant to online learning as trainees must stay motivated. We used independent sample *t*-tests to compare the average level of engagement in the feedback and non-feedback conditions and responses after part 1 and part 2. The results are presented in Table 5. These results indicate a higher level of engagement by the participants in the feedback condition; for both conditions, we found a slight increase in engagement between the first and second measurement point in the study. However, none of the results reached a level of significance.

#### 3.3.2. Quality aspects

In the context of developing a tool to practice communication skills for transfer to the professional field, the participants were also asked to evaluate the realism, consistency, and sensibility of the chatbot avatar and its conversational aspects. The results for the sample are presented in Table 6.

**Table 6:** Mean and standard deviation values regarding quality aspects of the avatar chatbot.

	Participants	After part 1		After part 2	
		Mean	SD	Mean	SD
Realism	30	3.77	0.76	3.61	0.64
Consistency	30	3.36	0.67	3.38	0.57
Appropriateness of the dialog	30	3.46	0.55	3.48	0.55

To assess the realistic quality of the avatar, we asked three questions on the degree of perceived childlikeness in turn-taking, the believability of the storyline, and whether the participants felt they were talking to a child. Scores on the three items representing aspects of realism were averaged.

The extent to which the participants experienced consistency in the story generated by the chatbot avatar also depended on how the chat sessions and chatbot avatar appeared to the users. Experienced consistency was measured using four items (as listed in Table 2) to map the experience of wholeness and internal logic to the story on a response level; see Table 6.

An assessment of the sense of appropriateness and the sensibility of the generated responses from the chatbot avatar, presented with a child’s name (Lisa) and a small image along with text just above it, showed that they were approximately the same across the two points of measurement and just above median level on the 5-point Likert scale. Perceived childlikeness was rated the highest after part 1 (*M* = 3.77). See Table 6 for the results.

#### 3.3.3. Learning experience

The perceived utility of the training tool was assessed at the end of part 2 only. Two statements were presented: (1) using the chatbot avatar aids me in acquiring knowledge and skills within questioning; (2) practicing with the avatar can enhance my self-efficacy. Independent samples t-tests were conducted. The results showed that, for the participants in the feedback and non-feedback conditions, the perceived usefulness of the chatbot for acquiring knowledge and skills had an average of *M* = 4.17 (*SD* = 0.84) and *M* = 4.17 (*SD* = 0.62), respectively.

For self-efficacy, the participants in the feedback condition obtained an average of *M* = 4.33 (*SD* = 0.78), whereas the participants who did not receive direct feedback reported *M* = 4.11 (*SD* = 0.58). The average score on self-efficacy was slightly better for the feedback condition, but there was no significant difference between the two conditions on either measure of experienced learning effect.

## 4. Discussion

In this study, we have demonstrated that online interview training that uses a self-run child avatar chatbot able to dynamically respond to an interviewer’s questions resulted in greater use of OEQs over multiple training sessions when combined with automatic direct feedback. The group of participants who did not receive direct feedback showed no significant learning effect regarding the asking of OEQs across the training sessions. However, the results showed that the students in the non-feedback group asked almost three times as many OEQs in the second interview session (after the first training and video lecture) as they did in the first chat session (which provided the baseline measure). This improvement, however, leveled out in the third interview session. Neither group showed a sizeable decrease in the posing of closed questions. The lack of decrease can be understood to indicate the complexity of the task of making interviewers (participants) prioritize the question category highlighted in the lecture (i.e., OEQ). OEQs were also in the first category of types of questions provided in the educational feedback that participants received. At the same time, given the level of detail necessary in a forensic setting, asking closed-ended questions might to some extent be a reasonable approach (Cyr et al., 2012). Another possible interpretation may be that interviewing, being a cognitively demanding task, may be too demanding for novices, making it difficult for them to make best use of the different question categories in the context of interviewing an alleged victim of child abuse (Griffiths et al., 2011).

Our findings partially support our first hypothesis. We expected to find an increase in the use of recommended OEQs by all the participants, but our expectation that there would be a decrease in the use of closed questions was not confirmed. The findings do verify the second hypothesis, revealing a significant increase in the use of OEQs and, thereby, an improvement in interview quality among the participants in the feedback condition. The participants who were provided with feedback were found to use significantly more open-ended questions than those in the non-feedback group in the third and fourth chat sessions, despite a 27.5% drop between the third and fourth chat sessions in the number of open-ended questions asked. These results confirm our third hypothesis that training paired with feedback is more effective in learning questioning skills than training without feedback. However, our findings indicate that one session of avatar training is insufficient. There was no learning effect in either of the feedback or the non-feedback participant groups between the second and third chat sessions, and there was a decline in the number of OEQs asked in the fourth session. The decline can be attributed to the one-week delay between sessions 3 and 4 and to the general lack of familiarity with OEQs. Previous studies have shown the effectiveness of multiple training sessions (and spaced practicing) with direct feedback offered immediately after training ends and, more importantly, have indicated the need for follow-up sessions months later to maintain interviewing skills in the field (Powell et al., 2016; Krause et al., 2017; Cederborg et al., 2021).

Overall, our results suggest that the automatic direct feedback function had an initial learning effect on the use of recommended (open-ended) questions. Being the first study to use our AI-driven classification model to code the interview questions in real time, our results both indicate the model’s efficiency and support further fine-tuning to achieve more detailed direct feedback. The finding that training alone is insufficient to produce a learning effect on the posing of questions as complicated as OEQs is in keeping with previous research results (Cyr et al., 2012; Yi et al., 2016; Cederborg et al., 2021) and the findings from studies on avatar use for interview training (Krause et al., 2017; Haginoya et al., 2020, 2023; Kask et al., 2022).

It is important to note that the learning effect observed in this study was seen even among the participants with very limited or no experience of interviewing children or of investigative interviewing in general. Considering the research that highlights the difficulties interviewers often face in translating theoretical knowledge from training courses to practical settings, the chatbot training tool could offer a superior learning effect. This tool might be especially beneficial for interviewers with considerable theoretical understanding but limited opportunities to apply their questioning skills and receive feedback in a practical context (Lamb, 2016). Continuous practice and feedback on performance are identified as important to maintain advanced communication skills. Incorporating direct feedback on questioning in an online chatbot-based interview training tool provides an available and flexible practicing opportunity. Our automatic classification model displayed a substantial level of agreement in the coding of types of questions within the three main question categories: open-ended, cued recall, and closed questions. We based our classification model on a large language model (LLM), GPT-3. This model performs considerably better than AI training, with automated feedback using n-grams as features and XGBoost as a machine-learning model, according to the research of Haginoya et al. (2023). LLMs can understand the context in which words appear; they are thus able to capture dependencies between words, phrases, and sentences. In contrast, n-gram models usually consider a limited context only, making them prone to missing important contextual information.

Future research should aim to handle detailed coding of various open-ended categories to be able to provide feedback and evaluate interview quality. This study’s feedback design is the first effort we have made to develop a feedback function, and it will be evaluated for improvement. One aspect to consider is the absence of a decrease in the use of closed questions. The variability in the pattern of questioning may illustrate the complexity of the skill of questioning, a lack of knowledge of or experience with interviewing children, or cognitive overload during interviewing that makes receiving and acting on the feedback difficult. In the feedback on closed questions that was provided, examples appeared on the screen last, and the closed-question category itself comprised a combination of types of questions. This may have made the feedback hard for the untrained interviewer to absorb.

With respect to the overall QoE and engagement, all the participants reported a positive experience while interacting with the chatbot. Contrary to our expectations, no significant difference appeared between the reported QoE of the participants in the feedback vs. the non-feedback condition. This may indicate that the participant interviewers perceived the chatbot to be a useful training tool. Engagement is well-established as a core factor in maintaining learners’ motivation and retaining them in courses (Lee et al., 2021). The participants’ reports of feeling engaged (i.e., losing track of time; being absorbed in the task) were higher among those in the feedback condition. A slight increase in engagement was found from the first to second measurement, indicating that engagement had the potential to increase over multiple child avatar chatbot training sessions.

Altogether, the ratings data on the quality aspects of the child avatar, which was presented with a child’s name and a small image along with text, were approximately the same at each of the two points of measurement (part 1 and part 2) and just above median level on the 5-point Likert scale. The perception of the avatar as childlike was highest after part 1.

The participants rated their learning experience with respect to knowledge and skill acquisition and self-efficacy. The ratings showed a positive attitude toward the child avatar chatbot. Self-efficacy, which refers to confidence in one’s own ability to perform in a specific context (i.e., to conduct an investigative interview), has been shown to be an efficient and reliable method for assessing the impact of communication skills training on professionals in the field (Nørgaard et al., 2012). As we used only one statement to measure self-efficacy, our results can be seen as indicative only. An expanded effort to measure this quality should be considered in future studies of professionals.

This study has several strengths. First, it offers empirical support for the potential of chatbot-based training tools to improve interviewing skills. Specifically, our findings indicate that a chatbot-based training tool can increase the use of OEQs, a recommended type of question with which investigative interviewers of children have traditionally struggled. Future research on the potential usefulness of this technology in professional domains such as law enforcement, child protective services, and mental health services could refine the chatbot’s capabilities, incorporate more realistic scenarios, and expand the sample size.

Several limitations must also be considered, including the small sample size, which affects the generalizability of the study results. Although we recruited broadly, a limited number of students signed up to participate in the study, which could indicate that those who signed up were particularly interested and motivated. Moreover, the representation of participants among the two conditions (feedback and non-feedback) was imbalanced. With respect to the patterns of questions asked, some of the interviewers (25% in the feedback group and 27.8% in the non-feedback group) interviewed the child avatar chatbot as if they were continuing an interview across more than one chat session (“So nice to see you again, Lisa”; “Do you remember what we talked about last time?”). This constitutes a limitation of the study as evaluations of real-life interviews suggest that conducting an interview over more than one session can lead to the asking of more cued recall and closed questions (Szojka et al., 2020). Any future study should provide clear instructions before each session about the context, or each interview session should assign a different name and/or scenario to the child that is being interviewed (virtually). Furthermore, a post-experience survey that posed more specific questions after each chat session might have better captured the interviewers’ experiences regarding the different aspects of the dynamic with the chatbot. Further studies should also include experienced interviewers among the participants to assess the transferability of learning effects to practitioners, not just those being trained in interview skills.

### 4.1. Conclusion and future directions

In conclusion, this study provides preliminary evidence that supports the use of self-run child avatar chatbots with an integrated feedback function in training for enhancing interviewing skills. Because the avatar is self-run, the flexibility with respect to when, where, and how often it is used for training is total. Dynamic dialog is a core interviewing feature when training communication skills. Further research will focus primarily on refining our dialog system to produce more authentic replies. To achieve this, we will use anonymized and pre-coded transcripts from real-life investigative interviews of children of various ages that are age-appropriate in all aspects and capitalize on the progress achieved in large language models (LLMs). We will undertake this in tandem with employing advanced techniques, such as neural radiance fields (NeRF) or generative adversarial networks (GANs), to create more lifelike avatars. Furthermore, we are focusing on augmenting the caliber of feedback and are investigating methods to optimally harness this feedback to enrich the comprehensive learning experience. Future research could further explore the potential of this technology in various professional domains, such as law enforcement, child protective services, and mental health services.

Complex skills such as interviewing need to be trained extensively and on a regular basis. This demands training tools, such as a child avatar chatbot, that evoke engagement—emotional, cognitive, and behavioral—and thus, future research into online learning environments would benefit from focusing on how to create and maintain engagement (Lee et al., 2021).

Furthermore, by leveraging technology and incorporating feedback mechanisms, educators can offer an engaging and effective learning experience that prepares students for real-world situations. More research is needed to optimize the chatbot’s performance and investigate its applicability across different contexts and populations.

## Data availability statement

The raw data supporting the conclusions of this article will be made available by the authors, without undue reservation.

## Ethics statement

The study received approval from the Norwegian Agency for Shared Services in Education and Research (SIKT), project number 614272. The participants provided their written informed consent to participate in this study.

## Author contributions

RR, GAB, SZH, SS, MR, MJ, and PH contributed to the conception and design of the study. SZH, SS, RR, GAB, and MJ organized the database. RR and GAB performed the statistical analysis. RR wrote the first draft of the manuscript. SZH wrote portions of sections of the manuscript. All authors contributed to the article and approved the submitted version.

## Funding

This research was supported by the Oslo Metropolitan University and Norwegian Research Council (grant #314690).

## Acknowledgments

We thank Lars Sokrates Anvil and Ingvild Olimb for their assistance in the collecting and coding of the data.

## Conflict of interest

The authors declare that the research was conducted in the absence of any commercial or financial relationships that could be construed as posing a potential conflict of interest.

## Publisher’s note

All claims expressed in this article are solely those of the authors and do not necessarily represent those of their affiliated organizations, or those of the publisher, the editors and the reviewers. Any product that may be evaluated in this article, or claim that may be made by its manufacturer, is not guaranteed or endorsed by the publisher.
